# Therapeutic potential of modulating endogenous PYY expression for controlling overweight and obesity: a narrative review

**DOI:** 10.3389/fnut.2026.1799041

**Published:** 2026-06-04

**Authors:** Zhijie Li, Junjie Yao, Sixian Wang, Jing Xu, Xingquan Wu

**Affiliations:** 1 Department of Acupuncture and Tuina, Changchun University of Chinese Medicine, Changchun, Jilin, China; 2 Department of Tuina, The Affiliated Hospital to Changchun University of Chinese Medicine, Changchun, Jilin, China

**Keywords:** appetite, dietary habit, massage, obesity, PYY

## Abstract

Endogenous peptide YY (PYY) plays a pivotal role in the regulation of appetite and body weight, and the upregulation of its expression provides a novel strategy for obesity intervention. This review systematically elaborates on the physiological functions of PYY, its secretory mechanisms, and the potential for promoting endogenous expression through multiple approaches. PYY is primarily secreted by intestinal L-cell. By activating NPY2R in the hypothalamic arcuate nucleus, it modulates the POMC/CART-NPY/AgRP neuronal circuitry and delays gastrointestinal motility, thereby reducing appetite. The synthesis and secretion of PYY are precisely regulated by multiple mechanisms, including nutrient sensors and nutrient transporters on L-cell. Dietary interventions, bariatric surgery, exercise therapy, and physical therapy can all upregulate Endogenous PYY levels by acting on L-cell or related signaling pathways, ultimately yielding benefits of appetite suppression and metabolic improvement. These intervention strategies mimic physiological pathways more closely, carry a lower risk of side effects, and may produce synergistic effects with conventional pharmacological agents. This article aims to synthesize current evidence, providing a theoretical basis and conceptual framework for developing individualized obesity management strategies based on Endogenous PYY modulation.

## Graphical abstract

**Figure:**
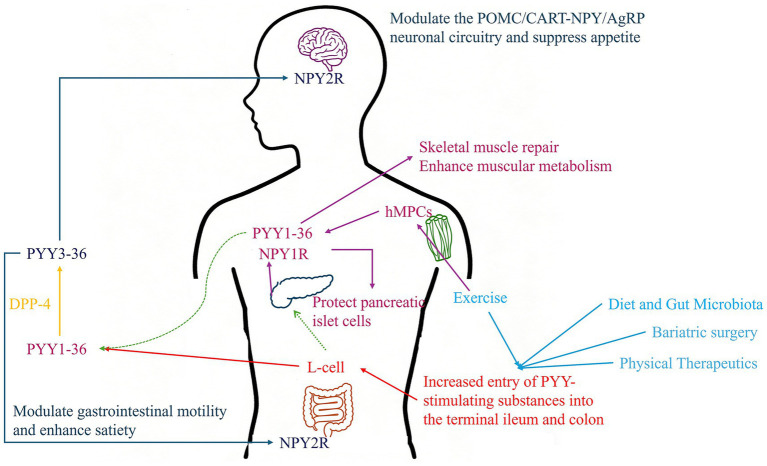

## 1 Introduction

Over the past few decades, the global prevalence of obesity has surged dramatically. Obesity has now emerged as a critical public health challenge confronting humanity, posing severe threats to individual health and socioeconomic stability. As early as the 1940s, the World Health Organization (WHO) classified obesity as a condition requiring intervention. By 2013, the American Medical Association (AMA) formally recognized obesity as a disease necessitating systematic medical interventions for prevention and treatment (1). Despite diverse approaches implemented to combat the obesity challenge, the World Obesity Atlas 2025 report by the World Obesity Federation (2) indicates that countries have failed to achieve their obesity control targets. Consequently, the number of individuals with obesity will continue to rise, and diseases attributable to overweight and obesity will pose escalating threats to human health.

The pathophysiological processes underlying overweight and obesity constitute complex phenomena influenced by multiple factors, including genetic predisposition, lifestyle habits, and social environment. Obesity can be classified based on etiology into primary and secondary obesity. According to metabolic status, it is categorized into metabolically healthy normal weight, metabolically unhealthy normal weight, metabolically healthy obesity, metabolically unhealthy obesity, and sarcopenic obesity. Pathophysiological phenotyping further divides obesity into four subtypes: brain starvation phenotype, gastrointestinal starvation phenotype, emotional hunger phenotype, and hypometabolic phenotype (3–5). These classifications reflect the complexity of obesity pathogenesis. Individual factors can independently drive obesity development through multiple pathophysiological mechanisms. For instance, abnormalities in genes such as *MC4R, LEP, LEPR, POMC, FTO*, and *SEC16B* disrupt appetite regulation, energy balance, and lipid metabolism, thereby mediating obesity onset (6). Concurrently, these factors interact synergistically to exacerbate obesity progression. Emotional eating triggered by anxiety and depression in daily life leads to hyperphagia and positive energy balance, ultimately causing obesity (7). During the storage of excess energy as triglycerides into lipid droplets, pathological alterations including endoplasmic reticulum stress and mitochondrial dysfunction occur, further aggravating lipid accumulation (8, 9).

This complexity poses significant challenges to obesity control efforts. Undoubtedly, however, the majority of patients exhibit pathological adipose tissue accumulation (10, 11). Body weight gain and adipose tissue accumulation fundamentally require caloric intake. Consequently, regulating absorbed calories enables body weight management, thereby addressing overweight, obesity, and associated comorbidities arising from these conditions. This concept is reflected in the therapeutic approaches for multiple obesity-related comorbidities (12–16). Calorie control can be achieved by inhibiting nutrient absorption in the digestive tract and reducing total caloric intake. Inhibition of absorption can be achieved through suppressing digestive enzyme activity, reducing the surface area for digestion and absorption, and shortening the residence time of chyme in the gastrointestinal tract; for example, Orlistat reduces fat absorption by inhibiting lipases in the digestive tract and has been used for obesity treatment since the early 21st century (17), while Roux-en-Y gastric bypass (RYGB) and One-anastomosis gastric bypass (OAGB) establish bypasses that allow food to circumvent portions of the digestive tract to reduce nutrient absorption (18). Reducing energy intake can be achieved by modifying dietary patterns to decrease total caloric intake, such as various weight-loss dietary models represented by the Mediterranean diet and low-fat diet (19, 20); it can also be attained through appetite suppression, exemplified by the use of glucagon-like peptide-1 receptor agonists (GLP-1RA) such as semaglutide (21, 22), or by performing bariatric surgery such as sleeve gastrectomy (SG) (23, 24). However, these existing intervention methods all exhibit significant limitations: for instance, Orlistat only inhibits lipid absorption and can cause severe gastrointestinal reactions (25); bariatric surgery is applicable solely to patients with moderate to severe obesity (23, 26) and is unsuitable for overweight individuals or those with mild obesity. In contrast, appetite suppression and reduced food intake can be universally applied to all patients requiring weight management. The recent upsurge in the use of GLP-1RA exemplifies this trend. In appetite regulation, GLP-1RA exert effects including appetite suppression, delayed gastric emptying, and enhanced postprandial satiety (27), leading to significant weight reduction in users. However, achieving the ideal weight loss efficacy demonstrated in clinical trials necessitates strict adherence to effective dosages with sustained use, which causes severe gastrointestinal reactions, markedly elevated risks of adverse events, and high treatment costs (22). Peptide tyrosine tyrosine (PYY) is a member of the neuropeptide Y (NPY) family. It is primarily secreted by intestinal L-cells and binds to Neuropeptide Y Receptor Y2 (NPY2R) on NPY neurons in the brain to regulate appetite and reduce food intake, while also exerting benefits in obesity and metabolism in other tissues (28). Compared to GLP-1RA, PYY exhibits a more gradual action and fewer side effects, while also reducing the required dosage of GLP-1RA (29), making it an ideal intestinal hormone for obesity treatment. Despite its numerous advantages, PYY has an extremely short half-life (30, 31). To achieve therapeutic efficacy, exogenous PYY formulations require frequent high-dose administration, which induces patient discomfort. Moreover, exogenous PYY exhibits variable efficacy across individuals (32), thereby limiting the development and clinical application of PYY-based therapeutics. Given these considerations, developing PYY analogues and NPY2R agonists represents a promising research direction to address these limitations. However, current PYY analogues face challenges including suboptimal efficacy and poor patient tolerance, restricting their clinical application. For instance, cellular studies by Ryan A. Lafferty et al. demonstrated that (P3L31P34) PYY1-36 and PYY1-36 (Lys12PAL) elicit beneficial effects on pancreatic β-cell function and survival comparable to native PYY1-36, yet with substantially reduced potency (33). Clinical trials conducted by Birgitte S. Wulff’s team revealed that PYY1875 can augment semaglutide therapy but exhibits poor patient tolerance (34). Meanwhile, NPY2R agonists such as BI1820237 and BI8271 require low-dose co-administration with GLP-1R agonists and have only recently entered Phase I studies in limited patient cohorts (35–37). Collectively, while PYY analogues and NPY2R agonists hold significant therapeutic potential, further refinement through continued research remains essential.

Recent years have witnessed profound advancements in related research, revealing that targeted interventions can upregulate Endogenous PYY secretion in humans. For instance, bariatric surgery has been shown to enhance PYY secretion in patients (38). However, bariatric surgery is typically indicated for patients with higher body weight or meeting other surgical criteria (39). For individuals not requiring surgery, particularly those with overweight or mild obesity, this approach to augmenting PYY carries health risks that outweigh potential benefits. Nevertheless, it undeniably establishes the feasibility of utilizing Endogenous PYY for appetite and obesity intervention. Increased Endogenous PYY confers multiple therapeutic benefits: centrally, it suppresses appetite and reduces food intake; peripherally, it lowers blood glucose and alleviates insulin resistance; additionally, for patients requiring concomitant GLP-1RA therapy, it reduces the required dosage. Beyond GLP-1, which is co-expressed with PYY, PYY itself ameliorates leptin resistance and insulin resistance that develop under conditions of obesity and metabolic dysregulation (40). This narrative review synthesizes research on PYY, discussing its physiological actions, secretory mechanisms, and the impact of current therapeutic interventions on Endogenous PYY regulation.

## 2 Physiological functions of PYY

PYY is a small peptide comprising 36 amino acids (41, 42), predominantly secreted by L-cell within intestinal enteroendocrine cells (EECs) (43). PYY belongs to the ancient NPY Receptor Family, originating from chromosomal duplications and differentiation hundreds of millions of years ago (44–46). Consequently, it can bind to multiple receptors within the NPY family and exert distinct physiological effects. A particularly well-characterized action involves binding to NPY2R in the hypothalamus, specifically the arcuate nucleus (ARC), resulting in appetite control and metabolic regulation (47, 48). Beyond L-cell secretion (49), sporadic reports indicate that PYY can be secreted in small quantities by specific neurons in the medulla oblongata and pancreatic *α*-cells, PP cells, and *δ*-cells (50, 51). PYY exists in two primary bioactive forms in humans: the full-length PYY1-36 and PYY3-36 cleaved by dipeptidyl peptidase-4 (DPP-4). These isoforms bind to distinct NPYR and exert divergent physiological actions (33). NPYR belong to class A G protein-coupled receptors (GPCRs), comprising distinct subtypes that selectively bind to NPY and PYY isoforms through differential recognition of their C-terminal and N-terminal domains (52). Humans express four NPYRs: NPY1R, NPY2R, NPY4R, and NPY5R. Depending on distinct bioactive forms of PYY they bind and their differential tissue distribution, these receptors serve distinct physiological functions.

### 2.1 Mechanistic basis and therapeutic potential of PYY in appetite regulation

The role of PYY in appetite regulation and its therapeutic potential constitute a major research focus in the field of PYY applications (28). Human feeding behavior is a volitional process broadly classified into homeostatic eating and non-homeostatic eating (including hedonic eating and stress-induced eating) based on the underlying drivers of food intake (53). Non-homeostatic eating leading to overconsumption is fundamentally distinct from homeostatic feeding. It involves the integration of cognitive, visual, olfactory, and hedonic cues by cerebral cortex regions, with food intake primarily motivated by pleasure-seeking. The insular cortex, anterior cingulate cortex, and orbitofrontal cortex accept these sensory stimuli and generate emotional responses based on cognition; these responses activate dopamine neurons in the ventral tegmental area, subsequently stimulate the striatum, link feeding behavior with pleasure attainment and stress relief, and establish a complex “reward system” that drives eating for hedonic gratification and stress alleviation (54–58). Homeostatic eating is primarily regulated by the hypothalamus, with key modulatory neurons distributed across regions including the ARC, paraventricular nucleus (PVN), ventromedial nucleus (VMN), dorsomedial nucleus (DMN), and lateral hypothalamic area (LHA), among which the ARC plays the predominant role. Within these brain regions, neurons co-expressing NPY and agouti-related protein (AgRP) in the arcuate nucleus (ARC) stimulate feeding, while those co-expressing proopiomelanocortin (POMC) and cocaine- and amphetamine-regulated transcript (CART) suppress feeding. The ARC serves as a primary hub for homeostatic appetite regulation. PYY3-36 from the gut activates NPY2R on NPY neurons, thereby modulating the activity of both neuronal populations (53). Other brain regions play a synergistic role, where CRH neurons in the PVN activated during human stress perception and SF-1 neurons in the VMN activated by leptin influence the activity of POMC/CART neurons and NPY/AgRP neurons, while the LHA exists downstream of NPY/AgRP neurons such that decreased NPY/AgRP neuronal activity reduces Orexin neuronal activity and consequently diminishes food intake (53, 59). Precisely based on this mechanism, peripheral signaling factors including leptin, oxytocin, and GLP-1RA converge on the hypothalamus, particularly the ARC, where they exert their regulatory effects (59). POMC neurons secrete the POMC precursor that is proteolytically cleaved to yield active peptides including *α*-melanocyte-stimulating hormone (α-MSH), which activates the melanocortin-4 receptor (MC4R) to achieve appetite suppression (60, 61). Generally, POMC expression in individuals shows minimal fluctuation in response to dietary factors; instead, appetite alterations are primarily driven by NPY/AgRP modulation of POMC pathways: AgRP competes with *α*-MSH for binding to MC3R/MC4R, while NPY suppresses POMC neuronal activity and directly stimulates feeding behavior (62). Under physiological conditions, this process enables normal hunger signaling, and equilibrium is spontaneously restored after adequate food intake. However, in obesity and other metabolic disorders, upregulated NPY/AgRP expression and disrupted feedback mechanisms lead to persistent hyperphagia (28, 63, 64). For instance, leptin and insulin inhibit NPY/AgRP activity while upregulating POMC/CART neuronal excitability (65–68); similarly, GLP-1 receptor agonists enhance POMC/CART neuronal activity (69). These mechanisms collectively modulate the interplay between POMC/CART and NPY/AgRP pathways to regulate appetite. However, limitations including frequent adverse effects of these agents and obesity-associated resistance to them, coupled with the chronic nature of obesity management, result in suboptimal benefit–risk profiles for appetite-suppressing pharmacotherapies. Consequently, PYY demonstrates substantial therapeutic promise. The impact of PYY on appetite can be summarized through three primary mechanisms: ① Modulation of the central POMC/CART-NPY/AgRP neuronal circuitry; ② Induction of satiety via gastrointestinal motility regulation; ③ Contributions from other potential factors. Generally, the effect exerted by PYY secreted from L-cell on the central POMC/CART-NPY/AgRP neuronal circuitry is considered the primary pathway through which PYY regulates appetite (53).

In summary, PYY has historically been regarded as a gut hormone, with primary research focus on the health benefits derived from appetite suppression, a perspective we endorse. However, due to the diversity and widespread distribution of NPYR, coupled with recent advances in research, novel insights have emerged regarding the specific mechanisms through which PYY influences appetite, obesity, and metabolism. Therefore, in the following sections, we will discuss the roles of PYY in these domains, categorized by NPYR subtypes.

### 2.2 PYY and NPYR

As previously indicated, the widespread distribution of NPY receptors (NPYR) across human tissues and the diverse bioactive forms of PYY contribute to significant complexity in mechanistic research. To address this challenge, we adopt a receptor-oriented framework—categorizing by NPY1R, NPY2R, NPY4R, and NPY5R subtypes—to systematically summarize tissue-specific localization and functions, thereby clarifying PYY’s targeted mechanisms and evaluating its therapeutic potential for obesity control.

#### 2.2.1 NPY1R: balancing appetite-stimulating risks and therapeutic opportunities

NPY1R is expressed in the cerebral cortex, thalamus, amygdala, as well as peripheral sites including adipose tissue, ileum, colon, and vascular smooth muscle cells (28). Owing to this ubiquitous distribution of NPY1R, the receptor exhibits distinct physiological values across different tissues in appetite regulation and obesity control, with PYY assuming diverse roles within these processes. It is generally accepted that NPY1R activation in the brain promotes appetite. For instance, NPY1R activation in the hypothalamic PVN modulates the reciprocal activity of AgRP and POMC neurons to promote feeding behavior (70). Furthermore, NPY1R in the brain mediates feeding behavior stemming from emotional regulation (i.e., non-homeostatic eating). Activation of NPY1R within emotional regulatory circuits induces anxiety and depression, thereby promoting hedonic eating (71). Therefore, blocking NPY1R activation in the brain can achieve appetite regulation. For example, using amphetamine to inhibit cerebral catecholamine release and downstream NPY reduces NPY1R activation, thereby suppressing food intake (72). However, current evidence does not indicate participation of Endogenous PYY in this process. NPY1R primarily binds intact PYY 1–36 with uncleaved C-terminal and N-terminal domains (73). Consequently, PYY1-36 secreted by EECs or other tissues is rapidly cleaved by DPP-4 into PYY3-36. Thus, NPY1R in the brain engages endogenous NPY rather than circulating PYY1-36. Theoretically, Endogenous PYY1-36 activating central NPY1R must derive from brain-derived secretion. Suppressing cerebral PYY1-36 secretion and NPY1R activation may inhibit appetite. However, mechanistic studies detailing brain-derived PYY1-36 activation of NPY1R in appetite regulation remain lacking. Although inhibiting NPY1R at this locus controls appetite, evidence defining the specific role of cerebral PYY secretion in NPY1R activation is absent. Thus, reducing brain Endogenous PYY1-36 expression to suppress NPY1R activation for appetite control lacks feasibility. These findings likewise inform the development of PYY-based therapies for appetite control and obesity containment. This association indicates that PYY-based therapies (particularly those elevating systemic PYY1-36 levels, including analogues) require avoidance of central NPY1R overactivation to prevent hyperphagia.

Outside the brain, NPY1R is also widely expressed and contributes to obesity pathogenesis, yet demonstrates minimal association with PYY. In adipose tissue, NPY1R is definitively associated with lipid accumulation, but exclusively linked to NPY, with no detectable PYY expression (74). In muscle tissue, NPY1R plays a distinct role. Studies demonstrate that NPY1R activation in the dorsal vagal complex (DVC) induces relaxation of gastrointestinal smooth muscle (75, 76), yet this effect lacks significant translational relevance for appetite or obesity management.

However, emerging studies reveal that modifications in skeletal muscle and pancreas following PYY1-36 activation of NPY1R demonstrate therapeutic potential for obesity control. Research demonstrates that during post-resistance training muscle repair, increased muscle progenitor cells (hMPCs) upregulate Endogenous PYY expression. Activating NPY1R enhances myofiber regeneration, and PYY secreted by hMPCs may enter systemic circulation, potentially exerting beneficial effects on metabolic regulation (77). In the pancreas, the absence of NPY2R and DPP-4 results in PYY1-36 being the predominant form secreted. This isoform activates NPY1R, protecting pancreatic *β*-cell and thereby exerting a positive impact on metabolic regulation (78, 79).

In summary, research on the physiological mechanisms of PYY1-36 and NPY1R reveals key considerations for developing and applying PYY-based therapies utilizing endogenous systems or exogenous agents: First, activation of central NPY1R must be avoided to prevent hyperphagia; second, PYY generated during moderate resistance training may exert systemic effects beyond muscle preservation and basal metabolic maintenance, for example through extra-intestinal net DPP-4 cleavage creating novel PYY3-36 production pathways; third, enhancing pancreatic PYY secretion protects islet β-cell function and associated insulin secretion. Furthermore, the actions of PYY and NPY1R in skeletal muscle and pancreas demonstrate therapeutic opportunities for PYY in addressing obesity and metabolic disorders.

#### 2.2.2 NPY2R: the central target mediating dual anti-obesity pathways of PYY

NPY2R represents the core therapeutic target for PYY-mediated appetite regulation (28). As noted earlier, PYY regulates the POMC/CART-NPY/AgRP neuronal circuitry by activating NPY2R in the hypothalamic ARC, representing the primary therapeutic target for PYY-based interventions (53). Beyond these sites, NPY2R is also expressed on vagal sensory neurons (80), in the spinal cord (81), kidneys (82), skin (83), as well as ovaries, adrenal glands, and joints (84, 85), with NPY2R on vagal neurons playing a critical role in satiety signaling for feeding control, while receptors at other sites exhibit weaker associations with obesity regulation.

Following expression of PYY1-36 by L-cell, it is rapidly cleaved by DPP-4 into PYY3-36. This truncated form exhibits remarkable stability in circulation and selectively binds to NPY2R (47). Structurally, PYY1-36 can theoretically bind to NPY2R but elicits markedly weaker activation compared to PYY3-36 (86, 87). Moreover, PYY1-36’s vulnerability to DPP-4 cleavage and rapid clearance from circulation prevent it from accumulating at sufficient concentrations to reach target sites. Consequently, this binding is physiologically negligible. Therefore, PYY3-36 serves as the most potent agonist for NPY2R.

As previously stated, NPY2R, which is predominantly localized in appetite-regulating regions including the ARC, directly suppresses appetite upon activation through modulation of the POMC/AgRP-NPY/CART neuronal circuitry (53, 88–90). Beyond central effects, NPY2R and PYY act synergistically in the gastrointestinal tract, reducing smooth muscle peristalsis to slow gastric emptying and intestinal transit, thereby promoting satiety. Due to the extensive distribution of NPY2R within gastrointestinal neurons, activation by PYY3-36 transmits satiety signals to the central nervous system, reducing food intake while preserving normal metabolic function (91, 92). Simultaneously, NPY2R in the mucosa and smooth muscle, when activated by PYY3-36, reduces ion transport at mucosal sites and inhibits muscle contraction in smooth muscle. These actions collectively delay gastric emptying and intestinal transit, enhancing satiety (93). Furthermore, building on these dual mechanisms, PYY demonstrates therapeutic potential for improving gastric tone, modulating gastrointestinal motility rhythms, and slowing chyme transit to stabilize postprandial blood glucose (29). Notably, clinical studies reveal that obese patients exhibit lower postprandial PYY levels compared to healthy individuals. The resultant insufficient activation of these processes contributes to hyperphagia and persistent feeding behavior, representing a significant factor in weight gain (94).

In summary, activation of NPY2R distributed in the ARC and gastrointestinal vagal neurons by PYY3-36 effectively controls feeding. This consistent mechanistic basis establishes NPY2R-targeted therapeutic development as the predominant strategy in PYY research.

#### 2.2.3 NPY4R/NPY5R: latent risks and suboptimal targets in PYY-based therapy

Unlike NPY1R and NPY2R, NPY4R and NPY5R can be activated by PYY, but current evidence remains insufficient to support their therapeutic value for obesity. NPY4R also possesses functions in weight reduction and metabolic improvement, but this receptor preferentially binds pancreatic polypeptide (PP), another member of the NPY family (95, 96). Although theoretically, PYY can activate NPY4R, and protein structure prediction, molecular docking simulation, and molecular dynamics suggest potential binding affinity (97), direct experimental evidence confirming its physiological relevance is lacking. NPY5R is predominantly expressed in the brain, where its activation by NPY synergizes with NPY1R to promote feeding behavior (98). In peripheral tissues, NPY5R expression is markedly lower compared to other NPYR subtypes (99, 100). In summary, NPY4R/NPY5R are suboptimal therapeutic targets for PYY-mediated obesity intervention. Nevertheless, this evidence holds significant implications for developing PYY-based appetite control and obesity containment therapies: specifically, preventing NPY5R activation-induced hyperphagia.

## 3 Secretory mechanisms of PYY

Overall, fluctuations in Endogenous PYY levels arise from various factor-mediated processes, including gene transcription, translation, and vesicular exocytosis. In terms of secretion sites, L-cell remain the primary source.

### 3.1 Regulation of PYY secretion: focus on L-cell and associated mechanisms

While L-cells constitute the primary source of circulating PYY, its secretion is modulated by a network of interacting elements. This section examines L-cell-centric regulatory mechanisms while acknowledging contributions from other EECs, nutrient transporters, and sensory receptors that collectively fine-tune PYY release.

PYY in human circulation is predominantly secreted by L-cell located in the distal ileum and colon, with secretory output progressively increasing from proximal to distal intestinal segments (49). L-cell express distinct nutrient sensors across intestinal segments and secrete different peptides in response to these signals (101). Collectively, the activation of diverse GPCRs on the surface of L-cell and their encompassing EECs by abundant luminal contents represents the principal mechanism for gut hormone secretion (102). Notably, free fatty acid receptors 2 and 3 (FFAR2/3, specifically GPR43 and GPR41) are definitively implicated in PYY expression (103). Additionally, the regulator of G protein signaling protein family (RGS), as negative regulators of GPCRs signaling, can participate in regulating Endogenous PYY secretion, thereby mediating appetite alterations and obesity development (104). Additionally, nutrient transporters and ion channels are also involved in regulating the secretion of Endogenous PYY (105).

Currently, receptors involved in L-cell release of Endogenous PYY include GPR19 (also known as Takeda G protein–coupled receptor 5, TGR5/G protein-coupled Bile Acid Receptor 1, GPBAR1), GPR40, GPR41, GPR43, GPR119, and GPR120, among others (103, 106). These receptors primarily promote Endogenous PYY release upon activation. These diverse GPCRs are capable of detecting a variety of substrates within the intestine, such as products of nutrient digestion and metabolites from microbial metabolism, among others. GPR19 is a GPCR that senses bile acids and their metabolites. It exhibits a higher affinity for secondary bile acids compared to primary bile acids. Upon activation by secondary bile acids such as Lithocholic Acid (LCA), it stimulates adenylate cyclase (AC), producing cAMP. This activates protein kinase A (PKA), promoting PYY release (107, 108). GPR40 is a Class A GPCR. Its intracellular C-terminus contains a palmitoylated cysteine residue and enables the receptor to bind Positive Allosteric Modulation agonist (AgoPAM) agonists while simultaneously recruiting both Gαq and Gαs. Gαs recruitment elevates cAMP levels, promoting PYY and GLP-1 secretion. Under physiological conditions, both midchain fatty acids (MCFAs) and long-chain fatty acids (LCFAs) can act as agonists for GPR40 (109, 110). GPR41 and GPR43 are GPCRs definitively associated with PYY secretion. Upon binding to short-chain fatty acids (SCFAs), they can activate EECs such as L-cells to secrete substances including PYY, GLP-1, and leptin. This cascade subsequently ameliorates conditions like obesity and metabolic disorders, establishing these receptors as classical and reliable therapeutic targets (111). Not only cAMP, but also Ca^2+^ drives the secretion of PYY. GPR93 is a member of the Class C GPCRs, specifically belonging to the calcium-sensing receptor (CaSR) family. Along with taste receptors such as taste 2 receptor member 5 (T2R5), it senses protein hydrolysates and incompletely digested protein fragments, respectively. Upon activation, it promotes the release of Gαq, leading to the activation of phospholipase Cβ (PLCβ) and the hydrolysis of phosphatidylinositol 4,5-bisphosphate (PIP2). This generates inositol triphosphate (IP3) and diacylglycerol (DAG), which in turn stimulate the release of Ca^2+^ from the endoplasmic reticulum into the cytosol. This calcium signal ultimately triggers vesicle exocytosis and the secretion of PYY. Furthermore, under conditions of prolonged stimulation, this pathway can counteract the obesity-induced impairment of pancreatic/duodenal homeobox-1 protein 1(PDX-1) binding to the *PYY* gene—an impairment mediated by RGS9 overexpression and hypermethylation—thereby promoting the transcriptional expression of PYY (90, 112). Activation of the CaSR by various L-amino acids—such as phenylalanine, tryptophan, aspartate, arginine, and glutamine—promotes the expression of PYY in L-cell (113, 114). However, not all CaSR activation events mediate PYY secretion to confer beneficial effects on weight loss. This process may occur in coordination with GPRC6a (115, 116). Furthermore, following the sensing of L-amino acids, metabotropic glutamate receptors 1 and 4 (mGluR1 and mGluR4) can elevating cAMP and cytosolic Ca^2+^ to promote PYY exocytosis (117). GPR119 is likewise a Class A GPCR. Upon binding to ligands such as oleoylethanolamide (OEA), it elevates cAMP and promotes incretin release (118). GPR119 is particularly sensitive to lipids and thus represents a highly promising therapeutic target (119, 120). GPR120 is also activated by MCFAs and LCFAs. However, to date, only the associations between GPR120 activation and weight loss as well as the upregulation of PYY expression have been reported. The precise role that GPR120 plays in obesity has not yet been definitively established (116). In summary, GPCRs present on the surface of L-cell, upon sensing substrates such as fatty acids and secondary bile acids, activate Gα proteins. This activation, in turn, engages cAMP and Ca^2+^ signaling pathways, along with their downstream transduction cascades, ultimately promoting the expression of PYY. As negative regulators of GPCR signaling, RGS proteins may serve to constrain the release of PYY. For instance, abnormally high expression of RGS9 in the intestine accelerates Gα inactivation to terminate GPCR signaling, thereby impeding the vesicular exocytosis of PYY, which may cause reduced PYY secretion leading to hyperphagia and ultimately contributing to obesity (104). Similarly, Secretogranin II (SCG2), a chromogranin involved in the packaging and sorting of peptide hormones, can promote vesicular exocytosis when its expression is upregulated. Studies have indicated that this upregulation leads to increased secretion of PYY and ameliorates obesity (121). In summary, L-cell GPCRs activate Gα proteins upon sensing substrates, engaging cAMP and Ca^2+^ signaling to promote PYY release. In contrast, the precise mechanistic details of how other factors—such as additional GPCRs, RGS proteins, and secretogranin II (SCG2)—participate in and regulate the release of PYY from EECs remain under active investigation.

Nutrient transporters can also be involved in upregulating the secretion of Endogenous PYY. The plasma membrane monoamine transporter (PMAT) and the serotonin reuptake transporter (SERT) facilitate the transport of metformin into L-cell. Upon activation, Adenosine 5′-monophosphate-activated protein kinase (AMPK) signaling is engaged, which ultimately leads to increased secretion of PYY (122). Beyond their role as nutrient sensors, these nutrient transporters can also exert additional effects to promote the expression of PYY. For example, Sodium-Glucose Transporter (SGLT) are responsible for glucose uptake. When inhibitors are used to suppress SGLT activity in the stomach and proximal intestine, more glucose is retained and delivered to the distal intestinal segments where L-cell are located. There, the subsequent signaling effects triggered by SGLT activation on L-cell, combined with the activation of other nutrient sensors by metabolites generated from microbial digestion of the retained glucose, act synergistically to promote the release of PYY (123–125). Furthermore, glucose can also promote the release of PYY through the activation of the gut sweet taste receptor (T1R2/T1R3) (126–128). Similarly, the glucose transporter (GLUT) family, which shares functional similarities with SGLT, is also capable of transporting glucose and other sugars into L-cell. This transport elicits Ca^2+^ influx, thereby triggering exocytosis and promoting the secretion of PYY (126, 129).

Similarly, evidence suggests that ion channels can also play a role in promoting the release of PYY. For instance, following its absorption into L-cells via amino acid transporters, L-valine metabolism leads to elevated intracellular ATP levels. This high ATP concentration closes ATP-sensitive potassium channels (KATP channels), thereby inhibiting K^+^ efflux. The resulting membrane depolarization activates voltage-gated calcium channels (VGCCs), which allows Ca^2+^ influx and ultimately triggers vesicular exocytosis (130).

In summary, the elevated concentration of circulating PYY derived from L-cell can be achieved via two principal mechanisms: enhancing its transcription and translation within L-cell, and promoting its vesicular exocytosis from these cells. In this regulatory process, a diverse array of GPCRs dispersed on the L-cell surface play a predominant role. Upon activation by the rich variety of substrates present in the gut lumen, they promote PYY secretion through second messenger-driven exocytosis. In contrast, RGS proteins act as a negative regulatory signal for GPCRs, inhibiting PYY release by attenuating GPCR signaling. Chromogranins such as SCG2, which are involved in peptide packaging and sorting, are suggested to play a restrictive role. Nutrient transporters and ion channels also function by transporting luminal substances into the cytosol, thereby initiating processes that stimulate exocytosis. Collectively, the pathways described above primarily modulate the circulating levels of PYY by regulating its release, without altering the total amount synthesized within L-cell. In addition, factors like LAT, PMAT, and SERT promote the transcription and translation of PYY via the activation of intracellular downstream signaling pathways, thereby increasing the total intracellular pool of the hormone.

Notably, receptors not traditionally linked to obesity and metabolic issues—such as mechanosensory like Piezo1 and immune receptors like pattern recognition receptors (PRRs)—may also have the potential to promote the secretion of PYY. Studies have shown that Piezo1 can sense mechanical stimuli from chyme passage and thereby promote the secretion of GLP-1 from L-cell (131, 132). Given that L-cell co-express GLP-1 and PYY, we postulate that mechanosensory like Piezo1 may also possess the capacity to stimulate PYY expression, although direct evidence is currently lacking. Furthermore, following the recognition of microbial surface components by PRRs, the activation of downstream signaling pathways such as cAMP/PKA or Ca^2+^-PKC may also promote the expression and secretion of PYY (103).

### 3.2 Expression of endogenous PYY in other anatomical sites

PYY is far more than just an intestinal hormone. As previously discussed in the context of NPYR, it exhibits (or may exhibit) local expression and secretion systems in several key organs, including the pancreatic islets, brain, adrenal glands, gonads, and skeletal muscle. Through paracrine and/or autocrine mechanisms, it acts as a complex and finely-tuned regulator in processes such as energy homeostasis, glucose metabolism, stress response, and reproductive function. Although current research suggests that the secretory systems in these sites may not substantially contribute to the overall circulating levels of Endogenous PYY, understanding the independent functions of this non-intestinally derived Endogenous PYY is, however, of significant importance for the development of therapeutic strategies aimed at stimulating Endogenous PYY secretion.

Within the pancreas, due to the absence of NPY2R, Endogenous PYY exists predominantly in the PYY1-36 form and signals through NPY1R. This signaling protects pancreatic β-cells and helps maintain islet integrity. Furthermore, it may promote β-cell replication by stimulating phosphoinositide 3-kinase *γ* (PI3Kγ)-mediated phosphorylation of extracellular-regulated kinase 1/2 (ERK1/2). Combined with its regulatory effects on *α*-cell, these actions collectively exert a positive influence on metabolic regulation (78, 79, 133). Similar to the outcome observed in L-cell following interventions, pancreatic α-cell and PP cell also exhibit increased expression and secretion of PYY. However, the precise mechanisms remain elusive and may be linked to circulating PYY derived from L-cell, as well as to the activation of receptors on L-cell by relevant substrates (134). Given that human pancreatic islets express only NPY1R, the circulating PYY3-36 isoform exerts limited effects. We therefore propose that the islets themselves, in response to incoming intestinal signals, activate the expression and secretion of PYY and concurrently suppress DPP-4 activity (135). Nonetheless, the increased secretion of PYY produced by the pancreatic islets remains derived from the stimulatory signals generated by the intestine following its sensing of diverse substrates. Direct infusion of PYY1-36 and DPP-4 inhibitors also appears to have therapeutic potential. However, to date, no studies have been reported on how to achieve targeted delivery. Moreover, as previously discussed, NPY1R/NPY5R signaling can promote appetite. Therefore, indiscriminately elevating circulating levels of PYY1-36 could potentially produce counterproductive effects on body weight control.

Within the brain, PYY itself also functions as a neurotransmitter involved in various physiological activities. However, whether the concentration of autocrinally secreted PYY in the brain can be modulated by external interventions to achieve the desired health benefits, or even whether the human brain itself expresses PYY, remains controversial. Although autocrine secretion of PYY has been detected in the brains of experimental rats, no evidence exists to specify the precise mechanisms, quantities, or physiological impacts of such secretion in the human brain. Consequently, the PYY that acts on NPYR in the brain is still believed to originate primarily from peripheral sources, notably the intestine (136). Similarly, the adrenal and gonadal glands may also be influenced by PYY. However, current research has not yet specified the precise mechanisms or physiological roles of autocrine secretion of PYY within these tissues and organs (84, 137–139).

From the current perspective, skeletal muscle may potentially contribute to circulating PYY levels. This is supported by observations that circulating PYY concentrations increase following both aerobic exercise and resistance training (140). Studies have suggested a possible association with the skeletal muscle repair process mediated by hMPCs. However, there is no direct evidence indicating whether this physiological activity within muscle can enter the circulation or what specific physiological role it might exert (77).

In this section, we have summarized current research on the expression and secretion of PYY. Undoubtedly, the secretion of PYY from intestinal L-cell into the circulation remains the primary research focus for developing therapies based on PYY. While evidence supports the expression of PYY within skeletal muscle and the pancreas, the underlying mechanisms are unclear. A key unresolved question is whether the increased secretion from these sites can transcend its local role as a neurotransmitter and enter the systemic circulation. Nevertheless, current findings suggest that modulating the autocrine secretion of PYY in these tissues may confer certain health benefits.

## 4 Mediating endogenous PYY expression: research progress

Based on the review of PYY receptors and secretion sites, it is evident that achieving health benefits through Endogenous PYY still requires interventions targeting intestinal L-cells. The potential contribution of Endogenous PYY from other anatomical sites remains largely theoretical. Currently, significant progress has been made in multiple therapeutic approaches for obesity, including dietary interventions, bariatric surgery, and pharmacotherapy.

### 4.1 Regulatory relationship of diet and gut microbiota on endogenous PYY

As elucidated previously, in the absence of exogenous agent administration, the quantity of Endogenous PYY is primarily determined by the response of L-cell to gastrointestinal contents—namely, dietary intake (whether from daily food or potential supplements). Furthermore, diet is inseparably linked to gut microbiota. The interplay between the human body, gut microbiota, and diet-derived gastrointestinal contents collectively represents the primary effector mechanisms governing Endogenous PYY production. We posit that this tripartite interaction modulates Endogenous PYY through three primary pathways: ① Direct Secretory Stimulation: Dietary components (including native constituents and their digestive metabolites) directly promote PYY secretion. ② Neuroendocrine Activation: Diet-induced acceleration of gastrointestinal motility increases distal intestinal delivery of bile acids, glucose, and other molecules, thereby activating chemoreceptors and mechanoreceptors to enhance PYY release. ③ Microbiota-Mediated Regulation: Diet-microbiota interactions reshape microbial community structures that potentiate PYY secretion.

From a nutritional composition perspective, proteins demonstrate superior efficacy over carbohydrates and fats in stimulating PYY secretion and appetite suppression, with both animal-derived and plant-based proteins exhibiting significant effects (140–142). Mechanistically, digestive amino acids (particularly L-amino acids) activate L-cell exocytosis of PYY (113, 114), while the linker for activation of LAT facilitates cytosolic leucine transport to potentiate PYY synthesis (90). High-protein diets concomitantly increase both total intracellular PYY stores and secreted quantities in L-cell, establishing protein as the most influential macronutrient for circulating PYY. Lipids and fatty acids contribute to PYY release through receptors like GPR40 upon sensing MCFAs and LCFAs (109, 110). Notably, high-fat diets pose health risks given that intestinal MCFAs/LCFAs primarily derive from dietary fat hydrolysis; merely increasing MCFAs/LCFA s intake consequently elevates caloric load. Nevertheless, lipids perform essential physiological functions beyond energy provision, necessitating regulated dietary fat inclusion. We therefore advocate ensuring daily essential lipid requirements while prioritizing sources with favorable nutritional and metabolic profiles (143–148). This context explains the research focus on SCFAs: unlike MCFAs/LCFAs, beneficial SCFAs are microbial metabolites (e.g., from *Lactobacillus*) of indigestible carbohydrates like cellulose, which confer multisystem health benefits beyond PYY elevation (149). Dietary modifications enhancing SCFAs production inherently associate with consumption of intrinsically health-promoting foods (150–152). Consequently, augmenting dietary indigestible carbohydrates (e.g., cellulose) yields significant advantages. Specifically, cellulose accelerates intestinal transit (153, 154), reducing apical sodium-dependent bile acid transporter (ASBT) uptake and promoting distal conversion of primary bile acids to secondary forms like LCA (155–157). It concurrently inhibits digestible carbohydrate absorption for glycemic control, while facilitating distal glucose delivery to stimulate PYY secretion (123–125). Mechanical stimulation via luminal content expansion directly activates L-cells to potentiate secretion (131, 132), collectively substantiating its therapeutic promise.

The interaction between gut microbiota and dietary components is critically important. Gut microbiota colonizing the entire gastrointestinal tract profoundly participates in human physiological and pathological processes, particularly in digestion, absorption, and metabolism. As repeatedly emphasized, gut microbiota significantly modulates PYY secretion. Microbial surface components, metabolic byproducts, and inter-microbial community interactions collectively regulate PYY secretion. For instance, SCFA-producing microbiota such as *Lachnospiraceae* convert indigestible carbohydrates into SCFAs, which activate GPR41/43 receptors to promote PYY secretion (158–160). Concurrently, primary bile acids are biotransformed by gut microbiota into secondary bile acids (e.g., LCA) that potentiate PYY release (155–157). Beyond microbial metabolites, microbial surface constituents and inter-species regulatory interactions also stimulate PYY secretion. *Lacticaseibacillus rhamnosus* strain HF01 exemplifies this dual mechanism: its surface components activate PRRs on L-cell, while its metabolic conversion of indigestible carbohydrates into lactate provides substrates for other microbes to ferment into SCFAs, subsequently activating GPCRs to enhance PYY secretion (103).

Current microbiome-based therapeutics encompass Prebiotics, Fecal Microbiota Transplantation (FMT), Probiotics, Live Biotherapeutic Products (LBPs), and Postbiotics (161, 162). Research on Prebiotics-PYY interactions typically employs efficacy metrics such as food intake, satiety, and serum Endogenous PYY concentration to evaluate specific Prebiotic effects. As exemplified by pectin and resistant starch-type 4 enhancing circulating PYY levels while modulating gut microbiota composition (163), and Berberine, Curcumin, and Galactooligosaccharides upregulating PYY expression (164, 165). Notably, Courteney C. Hamilton et al. report transient PYY modulation by Inulin-type fructans (ITFs) (166). Sina Maschek et al. demonstrate that specific gut microbiota in anorexia nervosa patients upregulate systemic PYY, inducing anorexic responses (167)—providing a rationale for utilizing FMT to establish stable PYY sources in appetite-regulation therapies, though no clinical studies have yet explored this approach. Analogous to Prebiotics research, Probiotics-PYY studies also rely on phenotypic biomarkers to assess efficacy, with mechanistic actions aligning with our prior discussions: microbial surface components and metabolites activate L-cell receptors to potentiate PYY secretion (119, 168–171). Postbiotics—encompassing microbial cellular components, metabolites, and derivatives (172) exert effects through previously described L-cell secretory mechanisms (173). LBPs conceptually represent standardized formulations integrating these therapeutic modalities (174), though no current applications target Endogenous PYY modulation.

In summary, specific dietary components are confirmed to stimulate PYY secretion, yet systematic analysis of current literature indicates no standardized, generalizable dietary protocol specifically for elevating Endogenous PYY levels has been established. We anticipate future research will enable comprehensive strategies to increase Endogenous PYY through approaches including receptor-targeted functional food design, microbiome-customized dietary regimens, and compound formulations that stimulate PYY release, among other methods.

### 4.2 Bariatric surgery

PYY benefits have been reported following bariatric procedures including SG, RYGB, OAGB, Adjustable Gastric Band (AGB), and Vertical Banded Gastroplasty (VBG) (18). These PYY alterations are fundamentally linked to gastrointestinal anatomical restructuring (175–177). Hypoabsorptive surgeries demonstrate the most pronounced effects, with RYGB exhibiting the most significant and sustained PYY elevation. Although Biliopancreatic Diversion with Duodenal Switch (BPD-DS) also enhances PYY, its high complication rate precludes mainstream adoption. Among restrictive procedures, SG shows modest PYY modulation (178). The impact of SG on PYY remains contentious: some studies report significant and persistent post-SG PYY elevation (179), whereas others find no statistically meaningful increase (180, 181). Mechanistically, considering L-cell secretion pathways and bariatric principles, we attribute greater PYY efficacy to hypoabsorptive techniques. Restrictive surgeries like SG primarily reduce gastric volume to limit food intake (182), exerting minimal direct effects on PYY. Compared to Gastric Banding or VBG, SG’s gastric tissue resection may influence PYY through intraoperative stimulation of NPY2R-rich neural plexuses during fundus removal (91), or altered digestive fluid dynamics redirecting metabolites to distal intestinal L-cell. Significant interindividual variability in SG-induced PYY responses exists (183). Discrepancies across studies likely arise from heterogeneous patient cohorts (e.g., diabetic status inclusion) or methodological differences (pan-ELISA vs. LC–MS/MS differentiation assays).

Based on previously discussed factors influencing Endogenous PYY secretion, bypass-constructing surgeries such as RYGB and BPD-DS represent the most compelling approaches for upregulating Endogenous PYY to control appetite and obesity. Key evidence lies in the direct delivery of chyme via bypass to the distal intestine, where protein/fat-derived substrates activate L-cell nutrient sensors and increase L-cell density (184). This facilitates the previously described secretory potentiation by bile acids and glucose (178, 184–188)^.^ Concurrently, altered luminal content remodels gut microbiota into configurations favoring PYY expression (189), with microbiota suppression shown to impair surgical efficacy (190). Furthermore, evidence suggests RYGB enhances pancreatic Endogenous PYY secretion through unidentified mechanisms, potentially involving secondary bile acid-TGR5 interactions (134).

In summary, hypoabsorptive bariatric surgeries—exemplified by RYGB—promote Endogenous PYY secretion by diverting nutrients to the distal intestine, where substrates activate specific receptors on L-cell. This process operates within established physiological pathways of L-cell expression. However, given current clinical indications for bariatric surgery, many overweight and mildly obese patients are not candidates for achieving Endogenous PYY upregulation through these procedures (191, 192).

### 4.3 DPP-4 inhibitors

We have previously elaborated on the physiological roles and secretory mechanisms of Endogenous PYY, along with the effects of various pharmacological agents; these will not be redundantly reiterated here. As established, circulating Endogenous PYY primarily functions in its PYY3-36 isoform, which activates hypothalamic NPY2R to exert anorexigenic effects. DPP-4 inhibitors offer a novel therapeutic paradigm: Agents like Sitagliptin inhibit pancreatic DPP-4 activity, thereby preserving PYY 1–36 levels locally. This enhances pancreatic β-cell function and promotes glycemic control, yielding significant benefits in diabetes management (134). Given the presence of NPY1R/NPY5R (appetite-stimulating receptors), developing pancreas-targeted DPP-4 inhibition holds clinical value. Indiscriminate elevation of systemic PYY1-36 may otherwise induce adverse effects—particularly paradoxical hyperphagia due to central NPY1R/NPY5R activation.

### 4.4 Exercise interventions

Exercise interventions can enhance endogenous PYY secretion. As noted in the section on PYY secretion sites, skeletal muscle repair mediated by hMPCs releases PYY^77^. However, evidence remains scarce regarding its systemic bioavailability and specific physiological effects. Most exercise-PYY studies focus on L-cell-derived PYY, employing phenotypic biomarkers (e.g., circulating PYY levels) as primary endpoints to evaluate therapeutic efficacy. Exercise consistently elevates circulating PYY levels and suppresses appetite (193). Critically, the temporal dynamics and functional outcomes of PYY secretion exhibit intensity-dependent divergence: light-intensity activities (e.g., walking, yoga) induce rapid PYY elevation within 30 min post-exercise, yet fail to acutely reduce caloric intake (194, 195); conversely, moderate-to-vigorous exercise elicits delayed but sustained PYY increases peaking at 45–90 min. These differential responses inform distinct therapeutic applications: light-intensity exercise synergizes with dietary interventions for immediate satiety potentiation and acute food intake reduction, while resistance training and moderate-vigorous protocols necessitate integration into structured lifestyle regimens to achieve sustainable metabolic benefits (194, 195). Given how exercise modalities promote PYY release, we propose that achieving sufficient exercise intensity may enhance PYY secretion. Although direct evidence in this field is currently unavailable, our hypothesis suggests that—when confounding factors are controlled—comparable activity volumes could stimulate PYY secretion across all populations, whether healthy individuals or patients with movement limitations, and regardless of movement type (voluntary exercise, assisted active movements, or passive activities). Based on this premise, exercise’s potential to boost endogenous PYY secretion applies not only to designing regimens for unimpaired individuals but also to mobility-restricted patients through tailored activity interventions.

### 4.5 Physical agent therapy

Physical agent therapy is a therapeutic approach that stimulates the body using physical agents such as electrical currents, light energy, and mechanical forces (196). Current evidence suggests physical agent therapeutics may modulate Endogenous PYY secretion, though mechanistic insights remain limited. Gastric Electrical Stimulation (GES)—involving electrode implantation in the stomach—paradoxically reduces circulating PYY levels (197), possibly mediated by electrically induced gastric contractions activating mechanosensitive ion channels or vagal afferent signaling indirectly suppressing L-cell activity via central nervous system pathways; however, no subsequent studies have substantiated these mechanisms. Similarly, conventional physical modalities (e.g., therapeutic ultrasound, electrotherapy) currently lack peer-reviewed evidence demonstrating clinically significant PYY enhancement, highlighting a critical knowledge gap in non-invasive interventions targeting Endogenous PYY pathways.

Researchers in traditional Chinese medicine have reported the efficacy of acupuncture and manual therapies in promoting Endogenous PYY secretion. Electro-acupuncture directly stimulates PYY release by activating the phosphatidylinositol 3-kinase/protein kinase B (PI3K/Akt) pathway, while concurrently upregulating tight junction proteins (ZO-1, occludin, claudin-1) and enriching *Muribaculaceae* (a major SCFAs producing taxon)thereby potentiating L-cell secretory function (198); abdominal massage upregulates the GPR41/GPR43-PYY/GLP-1 axis, significantly reducing food intake and body weight in high-fat diet-induced obese rats (199). Mechanistically, massage-induced GPR41/43 activation is mediated through structural remodeling of gut microbiota that elevates SCFAs production (200, 201), with abdominal manual therapy modulating microbial composition and metabolite abundance via glial cell line-derived neurotrophic factor (GDNF) signaling, nitric oxide synthase (NOS) activity, mast cell-regulated tryptase-PAR2-PKCε pathway, and interactions between tight junction proteins, intestinal barriers, and gut microbiota (202–204).

## 5 Discussion

This comprehensive review delineates the physiological role of Endogenous PYY in appetite and weight regulation, its secretory mechanisms, and the therapeutic potential of current interventions that elevate Endogenous PYY for obesity management. Substantial evidence supports Endogenous PYY upregulation as an effective anti-obesity approach, with well-characterized secretion pathways establishing circulating Endogenous PYY levels as a pivotal biomarker for therapeutic efficacy. While Endogenous PYY is increasingly recognized as a mechanistic target and holds significant promise in obesity therapeutics, formalized clinical protocols specifically designed for systemic Endogenous PYY upregulation remain underdeveloped.

PYY exerts its physiological effects through binding to NPYR, where despite the widespread distribution of NPYR, their binding affinities vary significantly across PYY isoforms and PYY expression is tightly regulated. Circulating PYY predominantly originates from intestinal L-cell, while skeletal muscle- and pancreas-derived PYY likely functions as local neurotransmitters with minimal systemic contributions. The core physiological significance manifests through L-cell secreted PYY activating hypothalamic arcuate nucleus NPY2R, effectively suppressing NPY/AgRP neurons and potentiating POMC neuronal function, concomitantly inhibiting ion transport and smooth muscle contraction to delay gastric emptying and intestinal transit, thereby enhancing satiety and suppressing appetite. Beyond appetite regulation, elevating Endogenous PYY confers metabolic benefits including amelioration of leptin resistance, glycemic control via reduced food intake and delayed nutrient absorption, with emerging evidence supporting intramuscular PYY-mediated metabolic optimization and pancreatic cytoprotection as promising research frontiers.

**Figure 1:**
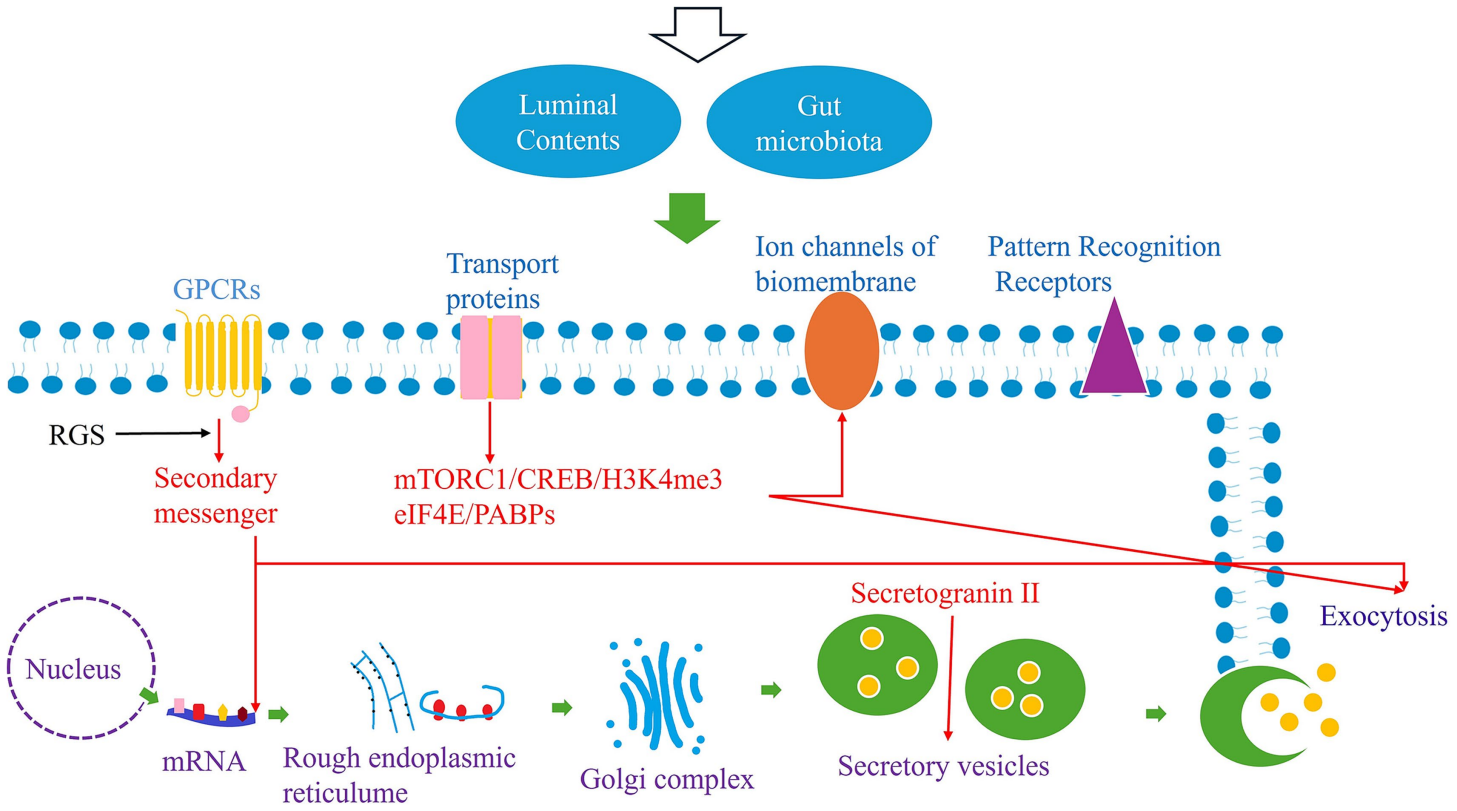
Intervention in the process of PYY secretion from L-cell.

Interventions targeting intestinal L-cell to enhance PYY transcription, translation, and vesicular exocytosis remain the most reliable therapeutic strategy, as circulating PYY predominantly originates from these cells. As shown in Figure 1, Numerous nutrient sensors—particularly GPCRs—upon activation, elevate second messengers to trigger PYY release. The regulator of RGS family, serving as negative modulators of GPCR signaling, may suppress PYY secretion when aberrantly overexpressed; conversely, upregulating vesicular packaging proteins like SCG2 potentiates exocytosis, identifying both as promising targets. Similarly, nutrient transporters, ion channels, mechanosensory, and immune receptors demonstrate significant efficacy in promoting PYY synthesis and secretion through parallel pathways. Current therapeutic approaches that elevate circulating Endogenous PYY concentrations primarily operate through receptor activation on L-cell surfaces. While pancreatic PYY arises from autocrine expression, its secretion exhibits strong associations with downstream signaling from intestinal L-cell receptors (e.g., TGR5 activation). In contrast, intramuscular PYY is exclusively generated during human muscle progenitor cell (hMPC)-mediated repair processes independently of gastrointestinal signals. Research evaluating these interventions universally employs phenotypic biomarkers—such as appetite suppression, weight reduction, and metabolic parameter modulation—as primary efficacy endpoints.

Dietary intervention undoubtedly constitutes the most fundamental and accessible approach. Within daily nutritional frameworks, increasing protein intake—whether animal-derived or plant-based—represents an effective strategy to stimulate PYY secretion. Lipids serve as physiologically essential components while their hydrolytic metabolites (MCFAs/LCFAs) directly potentiate PYY release; prioritizing lipid sources with favorable metabolic profiles over deleterious alternatives thus optimizes health outcomes without compromising physiological requirements. Similarly, elevating the proportion of non-absorbable carbohydrates (particularly cellulose) and slowly digestible carbohydrates (e.g., resistant starch) within total carbohydrate consumption shows significant promise. Nevertheless, translating PYY enhancement into precise dietary guidelines requires further systematic development. Beyond conventional diets, nutritional supplementation demonstrates feasibility through non-digestible carbohydrate supplements (e.g., cellulose formulations), microbiota-targeted supplements fermented into PYY-stimulating SCFAs, postbiotics directly activating L-cell secretion, and probiotic/FMT formulations. Although mechanistic and efficacy data exist for these potential supplements, standardized clinical protocols and mature formulations remain underdeveloped.

In bariatric surgery, malabsorptive procedures exemplified by RYGB induce anatomical reconstruction that diverts chyme directly to the mid-distal intestine. High-concentration substrates thereby activate multiple receptors on L-cell, concurrently increasing L-cell density and remodeling gut microbiota. Pancreatic Endogenous PYY secretion is also enhanced through intestinally derived signals, conferring islet protection and functional restoration. In contrast, restrictive techniques like SG demonstrate controversial PYY modulation—effects may derive from neural stimulation during gastric resection, accelerated chyme transit to distal gut segments, or interindividual variability—resulting in suboptimal efficacy and outcome consistency. For patients meeting surgical indications (moderate-to-severe obesity), bypass surgeries provide superior benefits in Endogenous PYY elevation and metabolic improvement. However, their cost-effectiveness diminishes in overweight/mild obesity cohorts, with limited evidence regarding PYY responses in these populations. For non-surgical candidates, developing supplements mimicking RYGB-driven microbial shifts—including prebiotics, probiotics, and postbiotics—holds significant research merit for PYY modulation.

DPP-4 inhibitors increase pancreatic PYY1-36 levels, activating NPY1R to protect *β*-cell and enhance islet function. However, systemic elevation of PYY1-36 may activate central NPY1R/NPY5R appetite-stimulating pathways, thereby limiting therapeutic applicability. Consequently, developing pancreatic-targeted DPP-4 inhibitors represents a strategy of greater research potential. Exercise interventions elevate circulating PYY concentrations, though the contribution of PYY released from hMPCs during muscle repair requires further validation. Exercise primarily modulates PYY secretion through L-cell regulation via gastrointestinal motility alterations, hemodynamic shifts, or indirect signaling cascades. Crucially, exercise intensity dictates differential outcomes: light-intensity physical activity demonstrates maximal efficacy for acute appetite suppression and food intake reduction. Notwithstanding, moderate exercise remains essential for overweight/obese individuals independent of PYY modulation. Consequently, exercise research should integrate with dietary strategies to establish comprehensive lifestyle interventions optimizing metabolic benefits. Physical therapeutics demonstrate considerable promise despite limited current evidence. Abdominal massage may enhance PYY secretion through mechanisms linked to gut microbiota modulation, intestinal barrier enhancement, and neuropathway regulation. Electro-acupuncture directly stimulates PYY release via PI3K/Akt pathway activation while concurrently modifying microbial composition and gut barrier integrity. These non-invasive approaches offer viable alternatives for overweight/mildly obese populations, though higher-quality clinical validation remains imperative. Furthermore, additional physical modalities exhibit therapeutic potential, urgently warranting rigorous investigation by domain specialists.

Augmenting Endogenous PYY expression represents a highly promising strategy for intervening in obesity and related metabolic disorders, with substantial existing evidence supporting this paradigm. Given PYY’s secretory dynamics and physiological actions, the foundational approach remains targeting intestinal L-cell to enhance PYY transcription, translation, and secretion into circulation, thereby achieving appetite suppression and reduced food intake. While pancreatic and intramuscular PYY secretion may confer ancillary health benefits, their mechanisms require deeper investigation. Current research predominantly leverages PYY as a phenotypic biomarker to evaluate intervention efficacy, reflecting well-characterized secretory pathways. Critically, therapeutic agents specifically designed to modulate Endogenous PYY remain underdeveloped. Future progress hinges on integrating dietary optimization, physical therapeutics, and surgical approaches to develop novel formulations and personalized multimodal regimens. Such combinatorial strategies hold significant potential for effective, sustainable management of overweight/obese populations.

For additional requirements for specific article types and further information please refer to “Article types” on every Frontiers journal page.
